# Being a top cop in pursuit of a sustainable lifestyle

**DOI:** 10.1080/17482631.2023.2235789

**Published:** 2023-07-20

**Authors:** Elin Granholm Valmari, Ulla Nygren, Mehdi Ghazinour, Kajsa Gilenstam

**Affiliations:** a Community Medicine and Rehabilitation, Umeå University, Umeå, Sweden; b Police Education, Umeå University, Umeå, Sweden

**Keywords:** balance in life, first responder, health, occupational patterns, patrol service, police officer, reflexive thematic analysis, roles, spare-time activities, work-life conflict

## Abstract

Previous research has widely recognized the challenges uniformed police officers face in their working lives. However, little is known about the overall lifestyles of police officers, including what they do in private life. We interviewed 17 officers and used reflexive thematic analysis to explore their experiences. The study sheds light on how uniformed police officers navigate the intersection between their private and professional lives, as well as how their professional role impacts their day-to-day doings and private life roles. The key findings relate to how the demands of their professional role impact their private life, such as the profession becoming a way of life. Furthermore, they highlight the challenges of avoiding certain environments where they might be recognized as police officers. It also entails balancing energy levels in work and private life, as well as how their profession’s unpredictability affects their daily routines and roles. The findings also show how their personal choices in private life are frequently influenced by their professional role. The study’s findings have theoretical as well as practical implications, contributing to a better understanding of uniformed police officers’ challenges and resources for a sustainable and healthy lifestyle.

It is widely acknowledged that uniformed police officers regularly engage in challenging contexts and environments. A uniformed police officer, as defined in this study, responds to emergency calls or engage in patrol duty within certain areas on foot or in a vehicle. They are also in daily communication with the public, keeping the public safe and upholding the law (“PATROL OFFICER, 2022; The Riksdag, 1998). Hence, the uniformed police officers adapt to a range of different crises, sometimes including hazardous and stressful work tasks during their working day (Granholm Valmari et al., 2022a; Mona et al., 2019; Violanti et al., 2017). These challenging contexts and environments have also been found to put a strain on their physical, social, and mental health (Granholm Valmari et al., 2022b).

Uniformed police officers’ demanding activities in work contexts and environments may also spill over into their personal lives (Qureshi et al., 2019). This may create a challenging social environment at home (Tuttle et al., 2018) while undertaking the role of romantic partner, family member, friend, or parent (Duxbury et al., 2021). However, research on police officers’ private lives, as well as research on the intersection between their work—and private life, is currently limited. Little is also known regarding police officers’ lifestyles. Therefore, this study addresses that gap by combining professional—and private life aspects of uniformed police officers, to explore the challenges and resources for having a healthy and sustainable lifestyle in all their contexts and environments.

## Police officers’ lifestyles and health

Existing research on the working lives of police officers has revealed the impact their jobs have on their mental (Demou et al., 2020; Violanti et al., 2017), as well as physical health (Magnavita et al., 2018; Mona et al., 2019). For example, police officers are at risk of developing cardiovascular disease, burnout, post-traumatic stress disorder (PTSD), and sleep disorders as a result of being exposed to stressful and traumatic situations regularly (Magnavita et al., 2018; Violanti et al., 2017). They are also prone to experience self-destructive lifestyle behaviours (Gershon et al., 2009), such as problematic drinking (Argustaitė-Zailskienė et al., 2020; Ballenger et al., 2011), hyper-aggressiveness, and violence both on and off duty (Beehr et al., 1995; Gershon et al., 2009). Furthermore, due to shift work and overtime, they have been reported to have a higher rate of suicidal ideation than others, not exactly clarified why (Violanti et al., 2008). In addition to this, a study on help-seeking behaviour within the Norwegian police force showed that anxiety was associated with seeking help for physical problems rather than psychological support (Berg et al., 2006). Moreover, role overload and work-life conflict have been studied in some countries but not extensively (Duxbury et al., 2021; Frank et al., 2022; Lambert et al., 2017; Li et al., 2018; Qureshi et al., 2019). For instance, work-family conflict and role overload were found to reduce job involvement and satisfaction among police officers (Frank et al., 2022). Contextual factors such as work culture and hours employed were also discovered to affect genders differently in terms of work-role and family-role overload (Duxbury & Halinski, 2018; Duxbury et al., 2021).

## Healthy and sustainable occupational patterns

Occupation is a term defined within occupational science (OS) and occupational therapy (OT) to explain the activities and tasks that people do in their daily lives that give meaning to life (Polatajko, Davis, Stewart, et al., 2013). Occupational patterns are created when a person goes about his or her daily occupations, structuring their habits in life (Wook Lee & Kielhofner, 2017). Occupational patterns are also influenced by contextual factors within individuals’ physical and social environments, including culture (Moll et al., 2015; Wook Lee & Kielhofner, 2017). However, it is not only contexts, environments, and occupations that are important in individuals’ occupational patterns; roles in life are also essential. For example, a person has one or several roles in each social environment. Roles also influence how people behave and interact with others. They shape what individuals do, divide daily and weekly cycles into time-use (Taylor, 2017), aid when forming habits (Wook Lee & Kielhofner, 2017), and foster a connection to others. Occupational patterns also reflect particular routines performed regularly, associated with social-cultural aspects (Polatajko, Davis, Stewart, et al., 2013).

Within OS and OT, occupational patterns are considered to reflect a person’s lifestyle (Matuska & Christiansen, 2008). A balanced lifestyle, also known as life balance, is defined as the need for biological health and physical safety, rewarding and self-affirming relationships with others, feeling engaged, challenged, and competent, and creating meaning and a positive personal identity. A person experiences life balance when they consider all these needs in life to be satisfactory. Additionally, the skill of organizing one’s time and energy is another aspect needed to achieve life balance (Matuska & Christiansen, 2008). A balanced lifestyle is considered sustainable (Matuska & Christiansen, 2008), as well as leading to health and well-being (Moll et al., 2015).

According to the World Health Organization (WHO), health is defined as physical, mental, and social well-being (World Health Organization, 2014). Health is also viewed as a resource for everyday life in the Ottawa Charter for Health Promotion (OCHP) (World Health Organization, 1986). It is considered to be more than just the objective of living, emphasizing the important link between people and their environments, the importance of a changing pattern of life over time, and how this affects peoples’ health (World Health Organization, 1986). To explain the impact occupational patterns have on health, five key concepts have been identified by Moll et al. (2015). Each concept; *engagement, meaning, balance, control/choice*, and *routine*, exist on a continuum, where optimal patterns lead to health benefits, while the other end of the continuum leads to potential health risks (Moll et al., 2015). Engagement refers to the initiating and sustaining of participation in particular occupational patterns. Meaning refers to the extent to which the patterns hold meaning for the person. Balance involves the nature and type of involvement, as well as patterns of time use. Control/choice refers to the degree to which a person perceives a sense of choice over their occupational patterns, while routines are regular and predictable patterns of behaviour or time use, including habits, rituals, and rhythms of life (Moll et al., 2015). Consequently, occupational patterns consider which occupations people do, as well as how they are engaged in them, and when and where people perform them. Hence, occupations can be health-promoting, such as different physical activities (Reiner et al., 2013) and social activities (Hyyppä & Mäki, 2003) which have been shown to have long-term health benefits. These same occupations can also be risk factors for ill health (Moll et al., 2015). Additionally, contexts and environments may also affect whether or not persons can engage in health-promoting occupational patterns and experiences (Moll et al., 2015). Hence, it is considered to be of great importance to identify both existing occupational challenges, as well as available resources regarding what enables a satisfying occupational pattern for a person (Polatajko, Davis, Cantin, et al., 2013).

## Rationale and aim of the study

As previously stated, different lifestyles among uniformed police officers present various challenges to maintaining a healthy and sustainable lifestyle. According to a recent systematic review (Granholm Valmari et al., 2022b), there is also a knowledge gap in what constitutes a healthy and sustainable lifestyle for uniformed police officers. While these police officers face challenges in their working lives, research on their personal lives, particularly their life balances, and imbalances, is lacking (Granholm Valmari et al., 2022b). It has also been previously suggested that the intersection between work and private life occupations of police officers needs further exploration (Frank et al., 2022). Therefore, we aim to investigate what enables a healthy and sustainable lifestyle, by exploring the challenges and resources that uniformed police officers experience in their occupational patterns.

## Method

The study follows an experiential qualitative framework to understand the perspectives of the study participants. Our fundamental approach is based on interpretivism to gather knowledge from the participants’ personal experiences and interpret the underlying meaning of their subjective realities. Thus, the study embraces a constructionist ontology and epistemology, acknowledging socially constructed, diverse subjective realities. Furthermore, we took a phenomenological stance, emphasizing the researcher’s connection with the study participants, and the creation of meaning through a process between the researcher and the participants. Thus, the study’s axiology values the participants’ lived experiences, where also values of the researcher merge with the research. Consequently, semi-structured interviews were chosen for data collection due to their ability to explore the participants’ experiences. Reflexive thematic analysis, according to Braun and Clarke (2013), was chosen to analyse the data and interpret the meaning behind these experiences, and understand the uniformed police officers’ lifestyles and health.

Braun and Clarke’s method is theoretically and methodologically flexible, offering an inductively oriented bottom-up procedure to capture both semantic (i.e., what the participant is communicating) and latent (i.e., interpreted meaning of what the participant is communicating) patterns of meaning. The method also allows for the use of relevant theory to connect findings to something broader (Braun & Clarke, 2013). Thus, theories from both OS and OT (Fisher et al., 2017; Matuska & Christiansen, 2008; Wook Lee & Kielhofner, 2017) were used to build the interview guide as well as to interpret the data after analysis.

To ensure transferability, the methods and results (including quotations) were described in full in accordance with the JARS-Qual (Levitt et al., 2018). JARS-Qual was also utilized to conduct the research and write the paper. Ethical approval for this study, as part of a larger research project, was obtained from the Swedish Ethical Review Authority on 4 February 2021, Reg No. 2020–07170.

### Participants and procedure

Sampling started in February 2021 and continued until August 2021. To ensure adequacy of data, firstly convenience sampling was used. Personally contacted police officers were asked to spread information about the project through posters, social media, and personal contacts within the Police Authority, even if they could not participate in the study themselves. Then, using snowball sampling during the recruitment process, participants were asked to help identify additional participants. Secondly, purposive sampling was used to get maximum variation and diversity regarding participants (gender, family situation, living and working in an urban or rural area, as well as locations in Sweden).

Participants who showed interest received information regarding the study (both oral and written), and an information letter was sent out, including an “approval to participate” form. A couple of people reached out but could not be included due to the study’s exclusion criteria; working or had recently worked within uniformed services. None of the researchers had any relation to the interviewed police officers before data collection.

The dataset included 17 uniformed police officers from the south to the north of Sweden, six of whom were women. They were between 29 and 51 years old, with a mean working experience of 8.7 years (ranging from 2.5 to 16.5 years). Some of them had also worked longer within the Police Authority, doing for example investigative work. The interviewed officers worked within different patrol services, for example, focusing on emergency response, road, or community policing duties. However, most of them worked with emergency response and because of that had a three-shift schedule. The participants with children had between one and four, from having small children to grown-ups, and not all of them had their children living with them all the time. See Table I for more information.

**Table I.** Participant characteristics as well as the interview topic guide.

Participants (Alphabetical listing)	Characteristics (Work information, family, and marital status)
Adam	Works in a small city or rural area and responds to emergency calls Cohabiting or married, and have children
Anne	Works in a small city or rural area and responds to emergency calls, Cohabiting or married, and have children
Ben	Works in a large city and responds to emergency calls Single or living alone, and have children
Charlotte	Works in a small city or rural area and works with community policing Single or living alone, and have children
Eric	Works in a large city and works with community policing Single or living alone, and have no children
George	Works in a mid-size city and responds to emergency calls Cohabiting or married, and have children
Greg	Works in a large city and responds to emergency calls Single or living alone, and have children
Jake	Works in a mid-size city and responds to emergency calls Cohabiting or married, and have children
Jason	Works in a large city and responds to emergency calls Single or living alone, and have no children
Kate	Works in a mid-size city and responds to emergency calls Cohabiting or married, and have children
Louise	Works in a mid-size city and responds to emergency calls Cohabiting or married, and have no children
Mary	Works in a mid-size city and responds to emergency calls Cohabiting or married, and have no children
Matthew	Works in a mid-size city and responds to emergency calls Cohabiting or married, and have children
Mike	Works in a small city or rural area and responds to emergency calls Cohabiting or married, and have children
Roger	Works in a small city or rural area and works with traffic Cohabiting or married, and have children
Sharon	Works in a large city and responds to emergency calls Single or living alone, and have children
Tomas	Works in a large city and responds to emergency calls Cohabiting or married, and have children
*Interview topic guide*	
*The interview guide centred around these topics, adding different follow-up questions depending on the person in front*: Work and private life roles, activities, and occupations; work duties, leisure activities, and other private life occupations and activities, including balancing of roles and occupations. Life roles, activities, and occupations in connection to global, local, and immediate contexts as well as the person’s social, physical, and occupational environments (including the temporal domain, such as scheduling, time use, etcetera). Values, meaning, engagement, and competencies in life, including positive and negative challenges in life. Health-related questions regarding physical, social, and mental health.	

A semi-structured interview guide was created by the first author and reviewed by the second and last authors. It was organized into topic areas reflecting aspects of a person’s occupational patterns. See Table I for the topic guide. Specific questions were utilized in the first few interviews, but following these interviews it was deemed more helpful to cover the topic areas by allowing participants’ narratives to direct the conversation, adding questions as needed. All interviews, however, began and ended with the same questions.

The interviews were performed in person at a place chosen by the participant, or via a visual feed, were audiotaped, and lasted on average two hours (ranging between 80 and 228 min). The interviewer (the first author) strived to encourage a relaxed atmosphere to make the participants feel at ease and receive their undivided attention. Also, the aim of the study and written information were repeated orally, and all participants were encouraged to make contact after the interview if additional thoughts or questions arose. A couple of participants made contact after the interview and wanted to add something.

The interviews were transcribed verbatim by professional transcribers, mostly a medical secretary, due to the sensitive nature of the content. The transcripts were proofread by the first author while checking for any deviation between recordings and transcripts. Emphasis or emotional expressions were noted in transcripts to ensure the transmission of intrinsic meaning and to strengthen trustworthiness. For reasons of confidentiality, identifying details were omitted or altered (Braun & Clarke, 2022).

After 17 interviews, the author group decided to stop the recruitment process as the richness of the dataset reached the point of information power (Braun & Clarke, 2022). The decision was based on the entire dataset of uniformed police officers’ occupational patterns, as well as participants included in the study (gender, work area; small, midsize, or large city, location in Sweden), and the length and quality of the interviews. Theoretical aspects of the study also informed our decision (Braun & Clarke, 2021, 2022; Malterud et al., 2016).

### Data analysis

A six-phase reflexive thematic analysis, including back-and-forth movement between the steps, was undertaken to explore the pattern of meaning across the dataset (Braun & Clarke, 2013, 2022). To establish trustworthiness, at least two, and sometimes all, authors were involved in several steps of the analysis. A reflective journal, including memos, was kept continuously while performing the interviews as well as analysing the data. These also included the researchers’ subjective impressions and thoughts, as well as feelings and problems encountered during the interviews.

The first author read transcripts numerous times, as well as listened to the interviews, and took notes in the diary or writing memos, to get a sense of the whole and become familiarized with the data. Furthermore, the entire author group was required to read and make individual notes, on a couple of interviews each. Following that, the similarities, and differences between each author’s notes were discussed, and a consensus was established on what the data included. This was done to assure the trustworthiness of the process’s familiarization step. During this step, we noticed a number of possible analytic areas of interest within the uniformed police officers’ occupational patterns, including the aim of this study.

Next, the first author used inductive initial coding, going line-by-line coding both semantic and latent meaning in the data relevant to a person’s occupational pattern. MAXQDA was used to perform the coding (VERBI Software, 2019). Coding involved repeated reading of the transcripts, looking for similarities, paradoxes, and controversy across the dataset. The second author also independently coded three of the interviews for purposes of reflexivity, providing additional standpoints. As the analysis continued over time, codes were refined and added to.

After coding, we set out to identify themes according to Braun and Clarke (2022). Firstly, the first author collated and clustered related codes into broad patterns of meaning. Secondly, clusters were discussed and sorted with the second author, and a pattern-based analysis using thematic maps was conducted. Several thematic maps were generated to demonstrate how participants made sense of their experiences across the dataset. It was also helpful in demonstrating how the topics overlapped as well as how they were connected and interconnected. Then, once there were distinct contrasts between ideas, including a central organizing concept, and the themes had strict limits between them, the first author developed candidate themes.

In the end, the first author repeatedly went back and forth between the themes, codes, and raw data, collating quotes for in-depth analysis. This was done to illustrate ideas and ensure that the analysis was firmly grounded in the data. As a result, the first author engaged in an iterative process of developing and refining themes. To ensure the trustworthiness of this process, the second author continuously took part in developing and reviewing these themes. To further strengthen the credibility, the results were also presented during a research meeting, where feedback was received from those not involved in the study. Following this stage, the patterns of meaning were presented and discussed by the entire author group. Having reviewed all the themes, the full dataset, and the coded data, we became more interested in the underpinning articulations related to what is described in this study, namely the overarching theme of “The forgotten self: Adaptability and duty above all”, as well as the two themes presented in this study. Therefore, we shifted towards developing this overarching theme, explaining the existing challenges and available resources of uniformed police officers’ occupational patterns for a sustainable and healthy lifestyle. Other facets of the uniformed police officers’ occupational patterns will be presented in other manuscripts. The entire author group reviewed the final analysis and ensured that quotations within a theme were consistent with the theme itself. Further clarity was sought to articulate ideas, as well as to illustrate and analyse the themes while writing up each theme as well as the report. When presenting the findings, fictitious names and quotes from all participants were used to show the grounding of data.

Periodically held extensive discussions between all the authors during the data analysis process resulted in the creation and finalization of themes. Each author contributed their knowledge, linking existing literature to the themes and expanding on patterns identified in the data, to provide a researcher’s perspective on the data and strengthen methodological integrity. The first and second authors are registered occupational therapists, the third is a social worker as well as a registered psychotherapist specializing in the mental health of police officers. The last author is a registered physiotherapist specializing in sports medicine, gender, and health. Throughout the entire process, authors with these different competencies and perspectives were involved in the analysis to triangulate, and thus increase, the credibility and dependability of the results.

## Results

**Figure 1.**
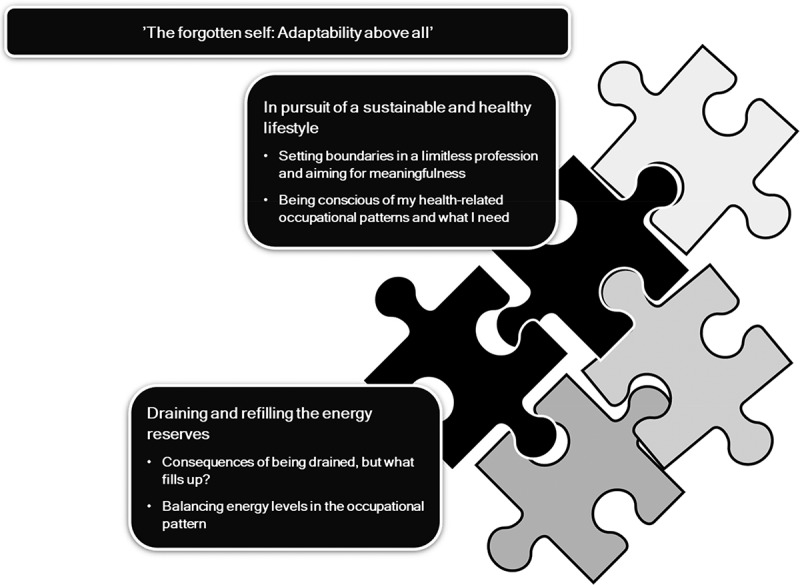
A conceptual map of the overarching theme as well as the two comprised themes.

***Note***. This illustration depicts the overarching theme as well as the two themes, which include second-order themes. The transparent jigsaw puzzle pieces represent additional facets of police officers’ occupational patterns that will be presented in other manuscripts.

A range of challenges and resources were identified pertaining to how uniformed police officers make sense of their lifestyle. The findings resulted in two themes (see Figure 1), “In pursuit of a sustainable and healthy lifestyle” and “Draining and refilling the energy reserves”. The two themes are incorporated in one overarching theme—“The forgotten self: Adaptability above all”. This overarching theme structures and adds contextual information to the two themes. See Table II for a summary of themes, including central organizing concepts and scopes.

**Table II.** A theme summary table including central organizing concepts and scopes.

Overarching theme	Characteristics
’The forgotten self: Adaptability, above all’	Includes how the uniformed police officers’ perception of constantly serving and protecting spills over and is also visible in their private lives. They adjust themselves at work to different situations related to the expectations of their professional role. However, they also adjust in other aspects of life; for example, if lines are not drawn clearly when leaving work, they continue adapting and continue to serve and protect at home, minimizing their own needs in order to have a sustainable and healthy lifestyle. Being constantly adjustable collides with having a sustainable lifestyle and the need to be “me”. Although the uniformed police officers are usually clear about what they need to stay healthy, the social group both at work and at home is usually prioritized before the self, but the reasons are different.
*Themes*	*Characteristics*
In pursuit of a sustainable and healthy lifestyle	This theme contains the ongoing struggle for health. It tells the story of the importance of being conscious of one’s own health-related occupational patterns and finding regular and predictable patterns. It also relates to which patterns are meaningful and what uniformed police officers need to stay healthy. The theme also includes the challenges and resources for setting boundaries in a limitless profession. Creating a sense of choice and control over one’s own occupational patterns is needed because work engages and spills over into private life. Thus, incorporating meaningful occupations within occupational patterns is expressed as important.
Draining and refilling the energy reserves	This theme includes aspects of constantly experiencing low energy levels and feeling tired or exhausted. It’s hard to get the energy to participate in private life occupations, whereas engaging in work-related occupations provides a better state of flow. The theme also contains aspects of which occupations drain and refill the energy reserves of the uniformed police officers, as well as what they experience as key factors when balancing energy levels.

### The forgotten self: adaptability above all

The overarching theme centres around both themes in the study as a contextual backdrop. First, it entails pursuing a sustainable and healthy lifestyle. Second, it includes the draining and refilling of energy reserves. Both themes include the adaptability to different work situations, a challenge as well as a resource for the uniformed police officers. Hence, adaptability is a key element to being flexible and responsive at work. They constantly move between challenging situations and unpredictable work assignments. Sometimes this is done simultaneously. Thus, being adaptable characterizes the uniformed police officers, adjusting themselves to meet the different situations within their social environment at work. However, it is an ongoing balance between the “self” and being a police officer during their overall patterns of time use. Consequently, it is easy to continue to “serve and protect” at home if roles are not separated when leaving work and physically, emotionally, and cognitively leaving the uniform in the locker. Moreover, unpredictability is a big part of their working lives, but when unpredictability occurs at home, the threshold of tolerance seems much lower. Hence, the energy needed for tolerance and adaptability seems exhausting and sometimes affects their social relations in their private life. Balancing these roles in life and finding sustainable occupational patterns to refill their energy reserves to participate in both private life and working life is a challenge.

Another challenging aspect is to distinguish themselves from their professional role. One reason is that uniformed police officers believe they are constantly scrutinized and recognized in society, such as when they go to parties, shop at the local supermarket, or visit gyms or restaurants. As a result, society’s perception of police officers as never truly being off-duty can make it challenging to find meaningful occupations outside of work. Also, the unpredictable work hours (due to shift work and specific work tasks) affect their occupational choices in private life. Consequently, they adjust their occupational patterns to fit the professional role and therefore struggle to maintain meaningful occupations outside work. By trying to take control of their occupational pattern, they instead reduce their opportunities to participate in certain occupations in private life. Making these adjustments is routine, and they don’t always register as a loss in life. Others, on the other hand, have successfully implemented resourceful routines to switch off their work mode, even if they continue to combine work and private life at home. Some have also found successful routines to balance the unpredictability of their work with private life occupations.

A resource for a sustainable and healthy lifestyle is that the uniformed police officers are very clear on what they need. For example, they describe the need for more physical activity and more debriefing at work. Additionally, being able to participate in meaningful occupations outside work is another example. However, job-related issues, such as an unpredictable schedule, make it challenging to pursue those requirements. Being constantly adjustable may also collide with their own needs. Especially since the collective, both at work and at home, is usually prioritized before the self. For instance, having a romantic partner and children at home presents a situation where neglecting one’s own needs involves prioritizing family care or experiencing guilt for lacking the energy to do so, or even both. At work, there is also a constant struggle to maintain their health and fitness to manage their physical workload. Hence, the struggle spills over into their private lives and interferes with their time off in order to fulfill their physical demands at work. Thus, “me” is easily forgotten when other demands present themselves. On the other hand, staying physically healthy is in itself not negative and is also what many of the uniformed police officers enjoy in life. However, incorporating health routines and predictable patterns of behaviour and time use into an unpredictable working life is a challenge, especially for those who are also parents. Consequently, the challenge often spills over to private life occupations and roles.

### In pursuit of a sustainable and healthy lifestyle

This theme contains the uniformed police officers’ constant pursuit of moving towards a sustainable lifestyle and health, including its existing challenges and available resources. Although many resources exist in this continuous pursuit, it’s a matter of locating them, as well as utilizing them efficiently. The theme centres around two subthemes: “Setting boundaries in a limitless profession and aiming for meaningfulness” and ‛Being conscious of my health-related occupational patterns and what I need’.

## Setting boundaries in a limitless profession and aiming for meaningfulness

This subtheme tells the analytic story of the challenges and resources encountered by uniformed police officers in their efforts to divide life into a private sphere and a professional sphere. The profession in itself never ends, and the service mode is always on, even if the interviewed officers don’t want it to be. Having blurred lines between different roles is a challenge, such as engaging in work when at home or out in society. The constant “serving and protecting”, engaging with work, is for some considered their calling in life. It is also considered their duty as an officer, or even a lifestyle that includes having their work as a hobby. Some interviewed officers have realized that they need to balance this calling in their private lives, for example through meaningful occupations or relationships outside work. However, it still seems to be a constant struggle, even for uniformed police officers who have been in the role for many years. As Greg puts it:

… well, there were quite a few off-duty interventions back in the day … and, of course, we do have a duty as well … But … you can’t just switch off and on. It’s also important to strike a balance, so you don’t find yourself driving around looking for work in your spare time …

It is our understanding from the interviews that this aspiration to engage at work, wanting to make a difference for others is important for the interviewed officers. This should be regarded as a resource since it provides meaning within their occupational patterns at work. They also hold themselves to high standards in their private lives, wanting to be able to look themselves in the mirror, both at work and at home. At work, they are driven by the need to deliver a good service to the public, to the people they serve, and want to make a difference for. At home, they try to live as an example to others. Thus, the police role tends for many to become a lifestyle, not just a profession. This however creates an imbalance in their own occupational patterns. As Jake says in answer to the question, “ … a way of life?”: “Yes, it’s very much a way of life, for me anyway. Because it’s what I expect of myself, that … a police officer is a police officer at all times … ”

Being a police officer at all times and in all roles in life makes it challenging to engage and participate in particular occupations in their occupational patterns. Greg explains this by also emphasizing that his profession impacts other occupations in his life: “But, of course, there are major consequences to this job; I avoid most restaurants, I don’t like to be out and about in town unless I have to … I mean, I place limits on my life because I work as a police officer … ”

Some of them also described that these consequences place restrictions on their families, and might be limiting, while others did not have this feeling. Additionally, another aspect was raised, when being off duty, everybody still sees them as police officers. This complicates their routines in their spare time. Being a police officer is also for some rather similar to having a higher calling in life, and for them, it is not always bothersome. However, even though it is not a higher calling for everybody, such as Anne—“just a job” – it does not make the line between the professional role and private roles any easier. Anne explains this as always being recognized as a police officer, never as “me”; and thus, being forced into her professional role other than the one she has on her days off when, for example, visiting a child playgroup:

It only takes one person to say you’re a police officer then everyone knows … I want to talk about diaper rash as well … I’m not interested in talking about my job … that’s not why I’m there … It’s my day off and I’m here for the kids so we can meet other parents and people don’t want to, they don’t get it.

Drawing these lines and balancing private and professional lives is a challenge. For Ann, being a police officer spills over into her private life. Even though Ann is trying to take control of her occupational patterns and set up proper boundaries between her work occupations and private life occupations. Despite this, she is forced into her professional role on her days off. Our understanding from the interviews is that for some of the interviewed officers, there is a constant struggle around also being judged as a police officer in their spare time. It is considered a challenge to switch between the professional role and private roles in their private life. This is not just because others see them as police officers all the time, but also because it’s difficult to let their guard down. As George says:

“ … when I took the garbage out, for example, I’d know that so-and-so lives in that apartment, and I would often run into them … I wouldn’t want to live there now because I would never be able to relax … In their eyes, you’re always police … I find that difficult”.

Another aspect of the importance of setting boundaries, as well as balancing private and professional roles, is that many of the interviewed officers attested to feeling as though the work might change who they are. For example, valuing life differently due to the amount of misery they see. Valuing life differently might on one hand be positive, having respect for life and seeing one’s own problems in life in relation to others’ problems. Nevertheless, witnessing a significant amount of misery might also result in them becoming cynical. Carrying an emotional backpack and what it might result in, such as cynicism also, worries many of the interviewed officers. As Matthew puts it when asked if he feels less emotionally affected nowadays compared to before:

… generally speaking, I don’t really care that much … but, yeah, numbed definitely … It’s, well, these days it makes me happy when I react emotionally to a case, because at least it shows that this means something, or this hit me particularly hard.

Many of the interviewed officers also find resourceful routines to deal with their emotional backpacks and to balance their professional and private roles successfully. Routines that were found helpful were having a long drive home after work, where they can listen to audiobooks or music and the mind is elsewhere. Others name carpooling with colleagues, where they can have casual conversations or vent during their drive home. Additional examples that were mentioned were keeping specific routines while switching between the uniform and private clothes. Another useful resource was the uniform itself, which many interviewed officers considered a shell for several things. It was regarded as a strategy for balancing the professional role with private life roles. As Roger puts it when asked if he has the time to rest and feel relaxed after work:

In principle, yes … I hang the job in my locker and go home. I think I have the right to do that.

Another challenge is that when the uniform is removed, many are still left with the emotional backpack, even if they are not always aware of it. Thus, wearing the uniform seems to result in an emotional overload instead. This is kept tightly locked at work but sometimes spills over to private life when taking the uniform off and going home. For Eric, it resulted in promiscuous living and regularly drinking too much to handle the bottled-up emotions. Ultimately, when his intimate relationship was being ruined it led to his realization that professional help might be needed. He, therefore, chose to seek advice from a counsellor, and learn new strategies to cope:

… I guess you could say I was a bit of a ladies’ man … anyway, I’d been seeing someone on the side behind my other half’s back. But I couldn’t really live with what I’d done. So, I chose to break it off and then I felt that I needed to talk about why I’d done it, with someone who was objective … I guess it was a way to deal with anxiety and stress … I wanted acknowledgement, to somehow feel better for a moment. A bit like alcohol … But there’s always a fucking hangover.

Many of the interviewed officers stressed the importance of having techniques and built-in routines for keeping responsibilities separated and balanced to avoid the police officer lifestyle or spillover from work into private life. Additionally important is finding ways to also empty their emotional backpack when the uniform is off, to avoid non-constructive ways of coping. Hence, balancing this aspect of work with private life is regarded as important for having a sustainable lifestyle. For example, the interviewed officers explain that having a lifeline somewhere in the social environment to hold on to is important. Balancing and comparing their worldview and occupational patterns at work with others’ experiences, for example, by having friends outside the authority, appear crucial. This also seems to help them find meaning in their occupational patterns outside of work, which sometimes could be a challenge.

## Being conscious of my health-related occupational patterns and what I need

This subtheme tells the analytic story of the importance of being aware of which occupations and routines the interviewed officers do and need to stay healthy. As well as how to balance occupations and different engagements in life so that their work does not spill over into their private lives in a harmful manner. As Ben explains it:

We are human, and we need to be seen, heard, acknowledged … I can’t come home feeling sorry for myself and pull away all the time. I have to work very actively on myself to make sure it doesn’t get the upper hand over my family. This job does affect both my wife and my children …

Here, Ben has found one key to experiencing sustainability—the ongoing project of trying to stay healthy by realizing how his occupations at work and his professional role might influence others in his family. Therefore, he constantly tries to reflect on himself. This is done to avoid letting his profession spill over into his private life, and not letting unhealthy routines manifest.

According to the interviewed officers, searching for emotional support in various ways is another aspect of being aware of the health-related occupational patterns and what the uniformed police officers require in life. Debriefing is offered at work after critical incidents, but it is rarely used in some workplaces. Debriefing is also decided by management, and police officers are sometimes not offered it for a variety of reasons. Sometimes they don’t realize they need it and decline the offer, or they hesitate to accept the offer from management due to the risk of stigmatization. As a result, many of the interviewed officers expressed a need for debriefing to be made mandatory after every critical incident, even if it was not desired at the time. The reason for this was that the need for debriefing doesn’t always occur directly after the event, but instead shortly after the offer has passed.

Besides debriefing, venting with colleagues is considered by the interviewed officers as an important factor for sustainable health-related occupational patterns. But having friends and families who give emotional support and understanding is also considered central, especially when communicating certain topics which at work might be taboo. However, neither of these types of social support, venting with colleagues or family members, seems to provide enough. Consequently, besides mandatory debriefing, many also suggest implementing regular professional talks or group coachings, such as by healthcare providers or police priests. In addition, many of the interviewed officers don’t even know how and where to seek help when needed. This makes the road longer than necessary if professional help is required. Health check-ups and more exercise at work were also aspects that were touched on and desired by the interviewed officers, to support and enable them to take control of their need for healthy and sustainable occupational patterns.

The irregular and frequently unpredictable work schedules, particularly during the night shift, also affect eating habits both at work and at home, making it difficult to maintain healthy and regular eating habits. Examples include simply being too tired to cook nutritious meals or findings time to eat at work. Another challenge is that police work is physically demanding, and many of the interviewed officers have experienced pain because of various physical interventions. Private life injuries, as well as age, may also have an impact when working as a uniformed police officer within patrol services for many years. Despite these injuries, the aspect of carrying heavy equipment at work is important to consider. Thus, the need for choice and control regarding individualized equipment should be realized within all regions, and should not differ, according to the interviewed officers. Roger explains his experience, where management was at first not very flexible, but then routines were changed regarding utility belts:

‟This whole process with drop leg holsters, that got very infected … it was extremely rarely granted, and then only with a doctor’s certificate … I just suffered in silence … my hip was getting more and more rotated. But when I finally made the decision about the leg holster, it was just a matter of signing out a new one from the service guy and then working with the weapons instructor to pass the test … So, I never needed to go to the trouble of getting a doctor’s certificate … ”

Working with pain appears to be normalized by some of the interviewed officers, albeit for various reasons. For example, some overlooked their own discomfort for reasons of safety. Charlotte explains why she doesn’t want to change her utility belt, although she would need to because of pain:

‟ … I’m scared because then my weapon isn’t sitting in the same position it’s always been … if I change its position, I don’t know if I’ll be able to find it in a stressful situation.”

The most visible component of being aware of health-related occupational patterns and what the interviewed officers require is the challenges and resources for maintaining mentally and physically sound habits. The interviewed officers mentioned physical activity to be one method to care for themselves both physically and mentally. As a result, they attempted to work out every day or at least several times per week. However, this is a struggle, and many believe that they do not have enough time to exercise to keep healthy at work or at home. One part is being overtired, whether as a result of the job, being a parent, or both. As Mary puts it when asked about the possibility of working out within the work schedule:

‟There’s no working out during working hours, except for one hour a week, and that’s outside the shift of course … it’s not like you can go off during the shift … if you perform patrol service, you need some form of exercise once a week, that’s how it is … officers in charge with staff responsibility might try to arrange for us to get together before a shift to play floorball, or whatever … ”

It’s not only the hourly workout that is important but also the matter of striving for daily exercise routines, according to the interviewed officers. Even when life is difficult and lacks regularity, finding exercise and exercise time that works for them within their occupational pattern was crucial. Kate who has a small child explains:

‟ … I try to arrange it so that, when he’s asleep, I can bring him with me skiing, in a sledge behind me … Then we go home and eat a snack and play until seven o’clock. Then we usually eat dinner together … Then we typically watch a movie and I usually fall asleep on the sofa at around half-past eight. By then, I’ve run out of energy.”

The interviewed officers agree that physical exercise is needed to stay healthy and that about three to five hours a week is needed in addition to the one hour received from work. However, the interviewed officers report that the amount of exercise performed varies greatly. The ability to adjust workout routines to factors such as family, shift work, and the availability of organized daytime training appears to affect the amount and type of exercise performed. Because of the unpredictability of their work, many of the interviewed officers work out alone or with colleagues.

### Draining and refilling the energy reserves

This theme centres around the amount of energy available to the uniformed police officers when engaging in occupations, roles, and routines in life. Many of the interviewed officers describe feeling exhausted at home, making it difficult to engage in everyday life, and giving that bit of extra to private life. The theme has two subthemes as follows: “Consequences of being drained, but what fills up?”, and “Balancing energy levels in the occupational pattern”. Eric describes the feeling of tiredness after work and how the drainage of his energy reserves feels from time to time:

‟I’ve felt that … how damn much of myself I’ve given … I’ve given a great deal … and I almost feel empty then … “

## Consequences of being drained, but what fills up?

This subtheme focuses on what the consequences are of having drained energy reserves, but also which occupations fill their reserves again. Work is what gives many of the interviewed officers the most energy. However, the effects of work, such as a lack of sleep or a lack of time to rest between shifts, can deplete energy reserves. It’s also what makes maintaining the social situation at home difficult. Mike who has kids says:

‟If I do a lot of evening shifts, I can feel really worn out, because then it’s all “go” at home until I leave for work, and then, when I get home again, it’s straight to bed. So, then I don’t get that break.”

Spare time activities are the occupations in private life where most energy is refilled. Physical activities of various kinds, as well as other more relaxing or practical activities, provide recovery. There are both challenges and resources for refilling drained energy reserves. However, the interviewed officers consider physical activity to be a main source for filling them. Louise adds an example:

‟ … everyone recharges their batteries in their own way. For some people, it’s being with friends, and I guess that’s true for me as well, it’s just that it’s so much easier to choose to stay at home, potter about in the garden or whatever because then it’s nice to enjoy the quiet. Not to have any demands … But then, in hindsight, I always appreciate being with friends or someone getting in touch or dropping by … but I never take that step myself because – for better or worse, I guess – I choose not to. I don’t see the downside until later.”

What we learned from the interviews is that engaging in meaningful occupations in life, aside from work, was a significant resource for replenishing depleted energy levels. Work should be balanced with meaningful and restful downtime to avoid energy drainage by continually “serving and protecting”. Meaningful spare time activities providing a sense of flow, such as engaging in sports or spending time with friends are described as examples that fill up their energy reserves. In addition, opportunities for relaxation are important. Adding to meaningful activities, it seems to be crucial to have time free from demands, since these occupations provide opportunities for relaxation. In summary, having a diverse social network as well as meaningful leisure activities seemed critical to developing a sustainable lifestyle. However, for many of the interviewed officers, work was the primary source of replenishing energy reserves, even more so than being at home. It is what Tomas calls a “safe haven”:

‟ … I guess that’s the only place … where it functions normally. I have my colleagues who are staunch friends, and I can leave everything else behind me. That’s why I really only feel anxiety coming on when I’m heading home … ”

Initiating and sustaining activities, chores, and obligations at home was another aspect when considering the difficulties of engaging in occupational patterns and the consequences of having drained energy reserves. Adam explains how he cannot muster the energy to do what is needed around the house, and what is needed to look after himself:

… I really, really like my job … somehow, I usually find another gear there but that has an impact later at home because then it’s a whole other thing … you might become irritated and feel, well … mostly I guess it’s a matter of not having the energy to deal with things … like cutting the grass and so on. You just don’t do those things; it’s more a matter of making it through the day … you do what you need to do but maybe not much more.

It is our interpretation of the interviews that the interviewed officers find their work to be so meaningful that they expend their entire energy reserves at work. This results in low energy at home. Therefore, it is important to discover sustainable occupational patterns also at home. Additionally, police work in itself is considered a central part of many of the interviewed officers’ lives, and one resource for providing them with energy. The reasons vary, but some examples are being where the action is, having good colleagues, meeting interesting people, and solving tricky situations. Greg adds to this by explaining why work gives him meaning, also in his private life:

‟Yeah, the small things … it doesn’t change the world (laughs) but it feels important to me … even if I’m involved in … a hell of a lot, of shit – if I can put it that way – it adds a whole other level of value to my life that I don’t feel other people get … for better or worse, you look at society with fresh eyes … so, even if it takes its toll, in some way I still think it’s been worth it. And then, I don’t place … such great demands on other parts of my life either.”

This meaningfulness at work may at times be paradoxical since it both provides meaning and may also overtake other occupations and roles in life. The consequence of over-engaging is for some of the interviewed officers the feeling of being drained of energy. This manifests in different ways, but for some, it causes sleep problems, or sleeping but not feeling recovered on waking. But it may also cause an imbalance in private life, such as not mustering the energy to live their everyday lives, for example finding the energy to do what is necessary for life, juggling different roles, such as being a parent, a family member, a friend, etc. However, Jason who lives alone also experiences this, he explains:

‟ … I can just stay at home in my apartment for two or three days without opening the door … And I’m super-sociable, so that’s not really me but … Work and all the rushing from place to place has given me an appreciation of how nice it is to just relax.”

Thus, being social at work and home both replenishes and depletes energy reserves. At work, it might be dealing with challenging citizens or co-workers; at home, for those who have children, it might be parenting. Work was also explained to be depleting in other ways, such as when constantly being exposed to challenging, uncomfortable, or tragic work assignments. Other examples were experiencing structural problems at work or feeling a lack of support from the Police Authority.

## Balancing energy levels in the occupational pattern

This subtheme centres around balancing energy levels in a profession that is influenced by unpredictability regarding time. The results indicate that having enough “me-time”, prioritizing one’s own health, and taking care of oneself is a critical aspect in maintaining a sustainable occupational pattern for the uniformed police officers. However, alone time and self-care is commonly disregarded by the interviewed officers who are also parents due to other responsibilities in life. Hence, parents must balance multiple roles and occupations in life, where children and chores normally come first, compared to those living alone. Another reason for limiting self-care and alone time is having a bad conscience as a result of frequently working overtime and not having enough time for family. Despite the difficulties, the interviewed officers emphasize the need for self-care, as well as dividing time alone when in a romantic partnership. Furthermore, having kids and finding time for oneself was an ongoing balance, and having personal control and choice over occupational patterns was not always possible in a romantic partnership or when having kids, although the need existed. But also, many felt so tired that it was also hard to muster the energy to participate in family and social life. Sharon had thoughts on how to balance a constant struggle between family life and taking care of her health:

… finding a good balance and getting to know yourself … That you learn things like … how should I sleep, how should I eat, how should I exercise and how should I recover so that I have enough energy.

When the interviewed officers do find time to prioritize personal occupations and self-care, it is usually through physical activities for the sake of staying healthy and because it is an unspoken requirement of their profession to stay fit. Although having time for physical activities was the main priority and concern for all interviewed officers to balance their energy levels, other meaningful activities were also raised. Mainly the importance of having practical activities, such as building for fun, doing garden work, spending time with animals, or repairing for example motorcycles. Another important aspect is finding occupations that clear their minds, or that provide the opportunity for letting off steam, which besides physical activity and practical activities, include socializing with friends. It is also important to consider that since the interviewed officers found physical activity to be a demand of the profession, not just a health benefit, finding complementary meaningful occupations that are just as engaging as physical activities were considered especially important.

The possibility to rest, such as to relax and wind down after work, was yet another aspect of their occupational pattern that was found to be important for a sustainable lifestyle in terms of balancing energy levels. Those without children or who were separated or lived alone found it much easier to balance their time towards relaxing activities, than those with families and children. Finding these personal routines or strategies for rest and sleep, especially when juggling children and shift-work, was important. Many of the interviewed officers had functioning individual strategies for balancing their energy levels and discovering them was what was considered important for a sustainable occupational pattern. For example, going back to sleep after leaving the children at kindergarten, as Mike does:

‟I’ve found a system that works … the night before I begin night shifts, I stay up very late but still get up early, so I get maybe five hours sleep that night … then I drop my son off at kindergarten and then sleep … I collect him then I go to work the night shift … when I get home in the morning … I sleep until the alarm goes off … it’s great.”

When the interviewed officers were unable to find well-functioning strategies for balancing energy levels in their occupational patterns, it was difficult to maintain the energy needed to engage in occupations in their private lives. This was due to all their energy being spent functioning at work. However, a good night’s sleep was naturally considered important. When not sleeping, their physical health was sometimes affected and different physical troubles manifested themselves, especially when the sleeping problems went on for some time. Thus, discovering resources within their occupational patterns that work for them, as well as their family situations, was crucial. One example of this was having an individualized shift system schedule at work. The interviewed officers with children who felt they have the resources for balancing their roles and occupations in private life also had a schedule that works for them and their occupational patterns. Sharon is one of those uniformed police officers:

‟I try to schedule work so that, as far as possible, I’m off when I have the kids and work as much as possible when I don’t have them. We have the type of schedules that allow us to plan our own working hours.”

However, for many of the other interviewed officers, their schedules were permanent or semi-fixed and couldn’t be adjusted. As a result, many of them mention sleeping problems or a lack of flexibility in their schedules while raising a family as reasons for abandoning their profession or leaving the three-shift system for alternative daytime work within the police authority. George, who has a family with children, explains how the work schedule can complicate routines in his occupational patterns:

‟ … If the contract had been different, it would have been considerably easier to make things work. As things stand, we have … eight hours of work and eight hours leisure time and eight hours sleep, roughly … when you have a job that often goes past those eight hours … and longer shifts at the weekend, it’s hard to make it work.”

According to the interviewed officers, the current work agreements, particularly the time compensation for working shifts and overtime, were also viewed as problematic. It was considered especially bothersome by the uniformed police officers with children.

## Discussion

### Summary of findings

This study has explored uniformed police officers’ challenges and resources for living a sustainable and healthy lifestyle. The article’s three key findings are police-specific challenges. Some of the challenges found are also in line with previously conducted research on police officers. Furthermore, additional findings for enabling sustainable and healthy occupational patterns are more general, such as the resources found in the study. As a result, the findings may be of interest to other first responders as well as anyone interested in work-life and occupational balance concerns.

*The first key finding* comprises the challenge of how working life influences private life, due to different reasons. Examples include the police profession being a lifestyle or a calling in life, or the requirement for police officers to intervene when witnessing crimes in private life. *The second key finding* adds to the challenge of how the profession influences private life occupations. The uniformed police officers avoid occupations and environments where they might be recognized as police officers. This is partly because of who they might meet, but also because they must explain and defend what they do. The unpredictability of their work is a *third key finding* that adds to the challenges. The unpredictability affects their daily routines in terms of family and social life, as well as eating, sleeping, and exercise habits, and their ability to rest and rejuvenate. The *main resources* discovered in this study were for example the importance of reflecting on one’s own occupational patterns to identify and balance occupations and roles in life, which contribute to and enable a sustainable and healthy lifestyle.

### Specific findings

The *first key finding* centred around drawing lines between working life and private life, including balancing occupations and roles. This was found to be a central challenge in the uniformed police officers’ lifestyle. Examples included balancing their life calling as well as their roles in life while maintaining a healthy private and working life. This balance appeared to be an ongoing challenge, as they were motivated by the meaning of their work, which sometimes gave them more energy than their private lives. However, the consequences of their work also depleted them because they expend all their energy on work. A previous study found that spending time caring for others regularly might cause risk factors for burnout and PTSD in first responders (Greinacher et al., 2019). On the other hand, according to Moll et al. (2105), meaning is also an important characteristic of an occupational pattern. As well as a subjective, individualized experience, according to Matuska and Christiansen (2008). This is in line with Shin et al. (2021), who conducted a study on work as a calling in life. Perceiving work as meaningful was found to lead to higher job satisfaction and lower levels of work-related mental ill-health for some employees. On the contrary, for others, this perception led to burnout. The conclusion was that organizational support is crucial in preventing the negative effects of perceiving work as a calling and instead promoting positive organizational outcomes (Shin et al., 2021). This is in agreement with Matuska (2012) who emphasizes that several needs are important for life balance and a sustainable lifestyle. As an example, satisfying mainly one need in life, such as creating meaning in life is not sufficient for life balance. Hence, time and energy must be invested in different needs in life to promote health (Matuska, 2012). Regardless of the reason, our findings indicated an excessive involvement in the police profession, both at work and in their personal lives. Thus, their profession was found to spill over into their private life occupations and roles. According to Moll et al. (2015), time spent in a state of flow may be linked to happiness and personal growth, but investing too much emotion in something might in turn lead to ill health. Hence, engagement in various occupational patterns is related to both health and illness (Moll et al., 2015). It was discovered in our study that maintaining a clear distinction between the police officer role and their personal lives is crucial for the uniformed police officers to lead sustainable and healthy lifestyles. However, this needed good strategies and routines to be able to balance roles and occupations in both working and private life. In accordance with Kumar and Kamalanabhan (2017) demands in life, or conflicts within the interface between work and family have been found to significantly influence burnout of police officers. Consequently, balancing occupations and roles, both in work and private life, in line with a person’s lifestyle is linked to health and well-being (Moll et al., 2015).

Many uniformed police officers in the study were also concerned that the police officer lifestyle would eventually affect who they are, even though they were adept at maintaining boundaries in their private lives. This is a legitimate worry given that trauma exposure in police officers has been linked to changes in brain structure and function (Shucard et al., 2012). Nevertheless, police officers in Sweden have a duty to intervene if witnessing crimes between shifts, according to Police regulations (The Riksdag, 1998). Hence, separating work from private life might produce occupational imbalance and role imbalance for police officers within their occupational patterns. This was something we also found indications of in this study. Previous studies on occupational imbalance (Håkansson & Ahlborg, 2018; To-Miles et al., 2022) and role imbalance (Marks et al., 2001; Stuart & Garrison, 2002) have been linked to ill health, supporting the linkage of perceived balance in life to health (Moll et al., 2015). Thus, balancing occupations and roles would be an important aspect to investigate further when researching police officers’ lifestyles and health. Accordingly, to promote a sustainable and healthy lifestyle, Matuska and Christiansen (2008) believe that sustaining a sense of balance in one’s life can help one live a more stress-free, healthy, and fulfilling existence.

The police officer lifestyle is also partly a consequence of the challenge of always being seen as a police officer in private life. This is the *second key finding* in this study. Hence, even if the uniformed police officers try to set up boundaries between work and private life, they are anyway considered to be police officers by the public. Always being recognized or viewed as a police officer while being in private life roles prohibited them from engaging in certain desired or meaningful occupations outside work. Consequently, the service mode was mostly on. Therefore, they avoided certain occupations and environments in their private life where they might be recognized, which also made an impact on their families. It also made them feel uncomfortable and made it difficult to let their guard down while doing private life occupations. This challenge to participate in meaningful occupations and roles within their private lives should be considered a risk factor. Similarly, Violanti et al. (2018) found that police officers who exhibited high social avoidance, such as avoiding public interactions, reported significantly lower social support. This “us versus them” mentality can be harmful to their health (Violanti et al., 2018). This is in line with the findings from this study. For example, signs such as the uniformed police officers’ change in worldview, cynicism, and feeling numb when dealing with work-related occupations, were discovered. Many of the uniformed police officers in the study attempted to offset this by releasing their emotional load through their social network, which should be considered a resource. It is also in line with Evans et al. (2013) who identified similar sorts of social support to be important for police officers. For example, supervisors, colleagues, and family members were considered a health resource (Evans et al., 2013).

*The third key findings* comprise the unpredictability of their work. The uniformed police officers in this study described that the unpredictability of their work occupations was important for filling their energy reserves because. They were considered meaningful as they included solving tricky situations, experiencing action and excitement, and meeting interesting people. In line with prior research on police officers’ personalities, action and excitement are frequently recognized as major factors in why police officers enjoy their work (Bowling et al., 2019). However, this study adds to the unpredictability of police officers’ work by how professional life is balanced with private life roles and occupations. The unpredictability of work, resulting in irregular routines, resulted in challenges regarding eating, sleeping, and resting. Balancing energy reserves was also found to come to a head in family life when organizing private life occupations. It was found important to recognize feeling constantly tired. As well as the spillover effect that using up all energy at work can have on one’s engagement in occupations and roles in private life. Thus, finding a way to balance private life occupations and roles with an unpredictable work situation was found important. This finding is in accordance with a previous study, where daily hassles of first responders were found to be significantly stronger associated with mental ill-health than the experience of traumatic events (Larsson et al., 2016).

Other police-specific challenges identified in this study for a sustainable and healthy lifestyle included the impact of carrying heavy equipment for an extended period of time. According to the Swedish Police Authority (2023), uniformed police officers carry between 10 and 12 kg of equipment every work shift (Polismyndigheten, 2023). This is not novel and has previously been studied in Sweden, particularly in terms of causing multi-site musculoskeletal injuries (Larsen et al., 2016, 2018; Ramstrand et al., 2016). Despite the Swedish Work Environment Authority’s ergonomics regulation for musculoskeletal disorder prevention, AFS 2022:2 (Swedish Work Environment Authority, 2012), individualized ergonomically correct equipment within the Police Authority is still lacking. According to Moll et al. (2015), the links between occupations in life, and health and well-being emphasize that what a person does during their day matters (Moll et al., 2015). Thus, stipulating that also strenuous physical load is an important risk factor for Police Authorities to consider in order to provide healthy and sustainable police officers.

The *main resources* discovered in this study regarding uniformed police officers’ possibilities for a sustainable and healthy lifestyle are similar to research found in other groups of people. For example, one important resource was the ability to discover sustainable and healthy occupational patterns by reflecting on one’s own occupational patterns. Another example was identifying which occupations refill energy reserves and which ones drain them. It was also found to be crucial to balance the energy reserves by engaging in meaningful occupations outside work, such as finding spare-time occupations that promote relaxation of both mind and body, building a rich social network, or performing practical tasks, while also prioritizing time alone. This is also supported by previous research on police officers, where spare-time activities have been found to be crucial to their well-being. Especially exercise was found to be positively and significantly related to a lower risk of burnout (García-Rivera et al., 2020). The present findings also add to previous research on police officers by indicating the importance of striving to balance challenging work occupations with meaningful private life occupations. As well as identifying routines and occupations in life that boost energy levels. This is considered important for promoting a sustainable lifestyle for uniformed police officers. The findings are also in accordance with Moll et al. (2015) regarding which occupational experiences in life offer possibilities for health, such as experiences that activate body, mind, and senses, connect with others, contribute to society, promote self-care, build security/prosperity, develop and express identity, develop capabilities and potential, as well as offer pleasure and joy (Moll et al., 2015). Accordingly, although challenging, the uniformed police officers managed to engage in at least some parts of these occupational experiences to promote sustainable occupational patterns. Engaging in these occupational experiences can be a way to find healthy routines and take control over one’s own occupational patterns. This promotes health and should be considered a resource for a sustainable and healthy lifestyle for the uniformed police officers. Hence, even though their profession is unpredictable and at times challenging, balancing both roles and occupations in life is possible. However, more research is needed into what resources in their occupational pattern are specifically important to lead a healthy and sustainable lifestyle for specifically police officers.

### Study limitations

The researcher is the primary tool for data collection and analysis in reflexive thematic analysis. The interviews and analysis in this study were conducted by the same person (the first author), which could be viewed as a limitation as well as a strength. As a result of being part of the research as a researcher, the prior knowledge of the researcher influences the analysis. Thus, especially the first author’s comprehension of the interviews had a substantial impact on data collection and analysis. Consequently, the understanding of the patterns found within the interviews may be limited, and essential features may have been ignored. To lessen the risk and provide a more complete comprehension of the material, the other authors participated in various stages of the study. Hence, many issues were discussed and analysed from numerous perspectives to enrich understanding and mitigate this risk. Furthermore, the goal of qualitative research is not to provide generalized facts, but rather, as in this study, to contribute with depth and nuances of uniformed police officers’ experiences.

Another limitation might be that the study did not apply member checking, such as when participants check the results for recognition and accuracy. However, due to the sensitive nature of the interviews, it would have been unethical to send transcripts out by email or post. We tried to minimize this limitation by confirming a summarized finished analysis with a couple of police officers not included in the study before the manuscript was submitted for publication. Thus, the findings were checked for recognition and accuracy and should be considered credible. Continuous field notes and a reflexive diary were also provided for the opportunity to check dependability throughout the study period.

A couple of limitations should also be illuminated regarding the dataset. Firstly, it mostly included uniformed police officers responding to emergency calls, working a three-shift system. Secondly, most interviewed officers were of Swedish origin, as well as were in heterosexual relationships. Consequently, this has affected the study results since a lack of transferability might be an issue. Furthermore, not all identified facets of uniformed police officers’ occupational patterns are presented in this paper. This might be considered a limitation as the entire interview should be seen as one entity, with the different patterns within the interviews intertwined. Hence, we chose to select a couple of patterns for a more in-depth analysis, focusing on the most prominent themes according to the aim of this specific study. We have also tried to compensate for this choice by using a well-described, structured analytic method, including rich descriptions of findings illustrated by quotes. The study context, for example, the participants and settings, has also been described as clearly as possible to improve transferability. It should however be noted that the study was conducted in Sweden, and the results should be viewed from that perspective.

## Implications for future research and practice

The findings from this study have both theoretical implications as well as practical implications. Theoretically, it adds to police health research by complementing existing knowledge. Thus, the result contributes to a greater understanding of uniformed police officers’ experiences of challenges and resources for experiencing a sustainable and healthy lifestyle. Moreover, a challenge to many uniformed police officers is navigating through the intersection between work and private life occupations and roles, indicating the importance of adding OS and OT to police health research. Additionally, this study also adds to previous research regarding the intersection between private life and working life not only concerning police officers, but also other first responders.

The findings also have several practical implications. It adds an in-depth qualitative perspective on what engages uniformed police officers, both at work and at home. The study indicates what is found meaningful to them in life, how they balance their occupations and roles, what their routines are, and what they need to take personal choices and control over their occupational patterns. Hence, the findings might be valuable to various areas of police education, preparing uniformed police officers to maintain or procure a healthy and sustainable lifestyle. The result may also be valuable to police authorities in relation to how they can support their police officers to have sustainable working lives.

In addition, the results provide the motivation to explore the clinical implications of the findings in a larger context. This is in line with Moll et al. (2015) arguing that promoting occupation, health, and well-being should always guide the development of tools that engage and empower people to reflect on how their time is being used (Moll et al., 2015). Hence, the development of a measure of prevention and intervention for uniformed police officers’ lifestyles is needed. Such an instrument could be a basis for screening and measuring theirs, as well as other first responders’ conditions for a sustainable and healthy lifestyle, which subsequently could spur the development of health promotion strategies using OS and OT scholarship.

## Bibliographic note

Elin Granholm Valmari- University lecturer and Doctoral student at the Department of Community Medicine and Rehabilitation -Occupational Therapy Unit. Occupational therapist.

Ulla Nygren- Associate professor at the Department of Community Medicine and Rehabilitation -Occupational Therapy Unit. Director of Studies, Head of Unit. Occupational therapist and specializes in occupational therapy research.

Mehdi Ghazinour- Deputy head and research leader at the Police Education. Research projects include police science with a focus on the police personality, stress, trauma, and working environment.

Kajsa Gilenstam- Associate professor at the Department of Community Medicine and Rehabilitation -Sports Medicine Unit. Physiotherapist and specializes in exercise physiology and sex/gender.
